# Preparation of a Series of Highly Efficient Porous Adsorbent PGMA-N Molecules and Its Application in the Co-Removal of Cu(II) and Sulfamethoxazole from Water

**DOI:** 10.3390/molecules28114420

**Published:** 2023-05-29

**Authors:** Shishu Sun, Xiaopeng Zhang, Yan Zhang, Tianyi Sun, Linhua Zhu, Zaifeng Shi, Dashuai Zhang

**Affiliations:** 1 Key Laboratory of Water Pollution Treatment & Resource Reuse, College of Chemistry and Chemical Engineering, Hainan Normal University, Haikou 571158, China; sss_1996@163.com (S.S.);; 2 School of Chemistry and Chemical Engineering, Nanjing University, Nanjing 210023, China; 3 Engineering Research Center of Biomembrane Water Purification and Utilization Technology of Ministry of Education, Anhui University of Technology, Ma’anshan 243032, China

**Keywords:** polyglycidyl methacrylate, polyamines, Cu(II) ions, sulfamethoxazole, wastewater treatment

## Abstract

This paper presents a highly efficient porous adsorbent PGMA-N prepared through a series of amination reactions between polyglycidyl methacrylate (PGMA) and different polyamines. The obtained polymeric porous materials were characterized using Fourier transform infrared spectroscopy (FT-IR), scanning electron microscopy (SEM), specific surface area test (BET), and elemental analysis (EA). Thereinto, the PGMA-EDA porous adsorbent exhibited excellent ability to synergistically remove Cu(II) ions and sulfamethoxazole from aqueous solutions. Moreover, we studied the effects of pH, contact time, temperature, and initial concentration of pollutants on the adsorption performance of the adsorbent. The experimental results showed that the adsorption process of Cu(II) followed the pseudo-second-order kinetic model and Langmuir isotherm. The maximum adsorption capacity of PGMA-EDA for Cu(II) ions was 0.794 mmol/g. These results indicate that PGMA-EDA porous adsorbent has great potential for application in treating wastewater coexisting with heavy metals and antibiotics.

## 1. Introduction

Water pollution is a significant global environmental issue, with harmful substances such as antibiotics and heavy metals being major components [1,2,3,4]. Antibiotics mainly originate from human and animal use and medical and industrial wastewater discharge [5,6,7]. The widespread use and discharge of antibiotics can increase their concentration in natural water bodies, affecting aquatic organisms and aquatic ecosystems [8,9]. Antibiotic residues can increase bacterial resistance and the spread of pathogenic microorganisms, posing a threat to human and animal health [10,11]. When released into the environment, the biological activity of environmentally persistent pharmaceutical pollutants may directly adversely affect non-target organisms, such as wildlife, and cause long-term impacts on ecosystem health and resilience [12]. Residues of various types of medicinal products (hormones, anti-cancer, antidepressants, antibiotics, etc.) have been detected in the various compartments of the environment, which raises the question of whether this represents a risk for exposed plants, animals and microbes, or for humans [13]. Reducing the use and discharge of antibiotics is one of the key measures to protect the health of natural water bodies [14]. Heavy metals can accumulate and enrich water bodies, as they are difficult to degrade and absorb by microorganisms and plants in nature [15]. When heavy metals exceed a certain concentration, they can harm the aquatic ecosystem [16]. Heavy metals such as copper ions, cadmium ions, and lead ions can cause varying degrees of harm to aquatic organisms and human health at certain concentrations, so measures must be taken to limit their discharge and treat water pollution [17]. Among them, copper ions are a harmful substance widely present in water bodies, toxic to aquatic organisms, which may cause death and imbalance in aquatic ecosystems [18]. Copper ions can also accumulate in organisms and lead to health problems [19,20,21]. An excess of copper ions in cells is detrimental as these copper ions can generate free radicals and increase oxidative stress [22]. Copper ions can also lead to the depletion of sulfhydryls, such as in cysteines or glutathione, which can also lead to the further generation of toxic hydroxyl radicals [23].

Heavy metal and antibiotic pollutants widely exist in various production and domestic sewage, and usually enter various surface water and groundwater at a certain residual concentration, causing pollution to water bodies. Because antibiotics usually contain rich functional groups, they can coordinate with heavy metals, which will further form complex pollutants, which will change the physical and chemical properties of pollutants. This increases the difficulty of governance, and the toxicology becomes more complex, which will pose a threat to environmental safety and human health. As such, finding effective treatment methods for the co-removal of heavy metals and antibiotics is crucial. Currently, the primary treatment methods include physical, chemical, biological, and combined approaches [24,25,26,27]. Physical methods, such as adsorption [28], ion exchange, precipitation, and filtration, have good removal efficiency for heavy metals [29] but are less effective for antibiotic contaminants. Chemical methods, such as oxidation, reduction, precipitation, and complexation, have good removal efficiency for heavy metals and certain antibiotics [30,31] but may produce by-products and secondary pollution risks. Biological methods, such as biosorption, bioreduction, biodegradation, and phytoremediation, have ecological, safe, and economic advantages and can play a significant role in treating antibiotic and heavy metal pollution [32]. Combined methods combine multiple approaches to take advantage of their respective strengths and achieve better treatment results. For example, combining biosorption and physical filtration can effectively remove heavy metals from water. Adsorption has received considerable attention due to its high efficiency, reliability, economy, and ease of operation.

Adsorption is a technique that involves adsorbents binding pollutants to their surfaces to remove them. There are various types of adsorbents, including natural substances and artificially synthesized materials. Adsorbents offer advantages such as efficiency, reliability, cost-effectiveness, and eco-friendliness in water pollution control [33]. Their surfaces contain active sites that can quickly bond with pollutants, and the adsorption effect is stable and reliable, unaffected by other chemical substances and environmental factors in the water. Adsorbents are inexpensive to prepare, easy to manufacture and handle, and do not cause secondary pollution, resulting in minimal environmental impact [34]. Adsorption has been widely used in various water pollution control applications [35]. With the continuous emergence of new types of adsorbents, the importance and role of adsorption in water treatment will become increasingly significant in the future.

The preparation of highly efficient adsorbents has significant value and promising prospects for solving water pollution problems. Poly(glycidyl methacrylate) (PGMA) is a commonly used adsorbent material with an epoxy functional group, which facilitates various modifications [36]. PGMA was used as the substrate because of its known good mechanical strength and high reactivity of the epoxy groups for surface grafting. In addition, the PGMA beads have already been used in columns of chromatography in the industry. The amine group is one of the most effective functional groups for the removal of heavy metal ions; modification of PGMA with polyamines can increase the number of adsorption sites and enhance its adsorption performance [37]. During the adsorption process, the amine (-NH_2_) groups on the adsorbent can be complex with Cu(II), and during the co-adsorption process, metal ions can provide bridging sites for antibiotics, increasing the adsorption capacity of the adsorbent. In this study, a series of experiments and tests were conducted to validate the high adsorption performance and stability of PGMA-N macroporous adsorbent for harmful pollutants in water. This research provides new ideas and methods that can contribute to the field of water pollution control, protecting aquatic ecosystems, and human health.

## 2. Results and Discussion

### 2.1. Characterizations

**Figure 1:**
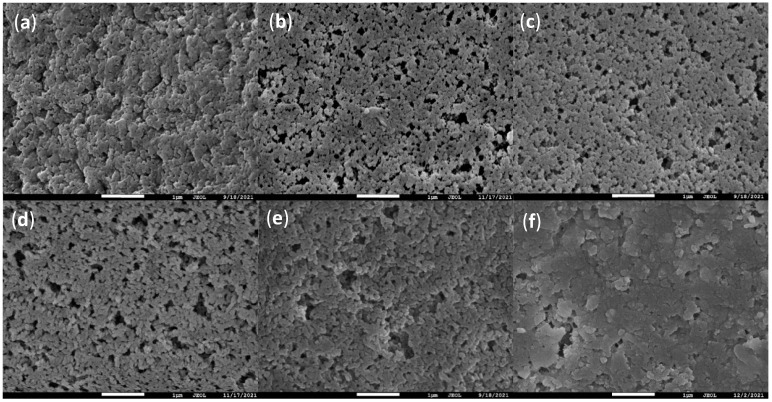
SEM images of PGMA-N series adsorbents: (**a**) PGMA; (**b**) EDA; (**c**) DETA; (**d**) TETA; (**e**) TEPA; (**f**) PEI.

SEM images can reveal the morphological features of adsorbents, such as surface roughness, porosity, particle size, and so on. These characteristics affect the contact area and interaction force between adsorbents and pollutants. In general, adsorbents with higher surface roughness, porosity, and smaller particle size exhibit better adsorption performance. As shown in Figure 1, (a) PGMA polymer spheres are uniform and smooth spherical particles without an obvious porous structure. They may have a low surface area and weak hydrophilicity; therefore, they are not suitable as effective adsorbents. (b)–(f) Adsorbents modified by ethylenediamine and its derivatives all exhibit irregular and porous particle morphology. They have a high surface area and strong hydrophilicity; therefore, they can interact with more pollutants in water. The above description of hydrophilicity is obtained after the contact angle test. The contact angle of PGMA-N before modification was 55.8°, and it became significantly smaller after modification. Among these modified adsorbents, (b) ethylenediamine-modified has the highest surface roughness and the smallest particle size. This may be the reason why it has the best adsorption performance. While (c)–(f) with the increase in ethylenediamine derivative chain length, both surface roughness and particle size decrease; therefore, the adsorption performance also weakens. In particular (f), the PEI-modified PGMAs seem to lose their microsphere structure and instead appear aggregated, which is one of the reasons for their lowest adsorption capacity.

**Figure 2:**
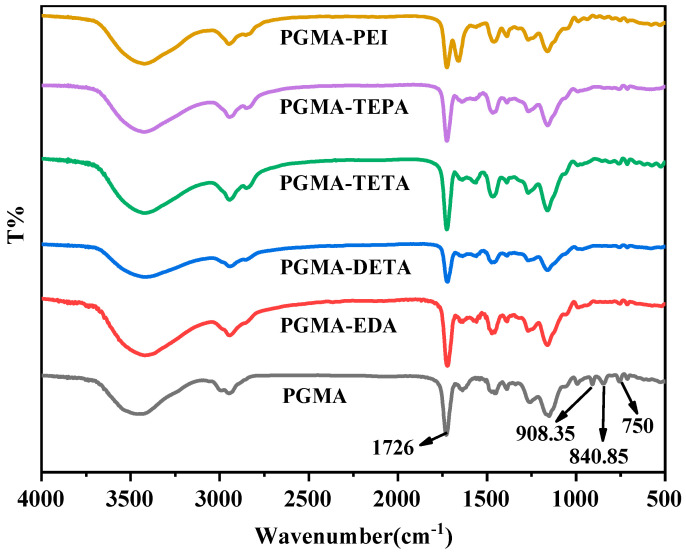
FTIR images of PGMA-N series adsorbents.

To investigate the surface modification effect of polyglycidyl methacrylate (PGMA) microspheres, this study characterized PGMA microspheres modified with different modifiers using infrared spectroscopy (IR) technology. As is shown in Figure 2, the following points can be analyzed: Compared with PGMA, the asymmetric stretching vibration peak of the epoxy group at 908.35 cm^−1^ of other modified adsorbents disappeared, indicating that the epoxy group reacted and was successfully opened. The carbonyl peak at 1726 cm^−1^ weakened with the increase in amino group number, indicating that carbonyl and amino groups reacted. The C-H vibration peak of PGMA is located around 2900 cm^−1^. After modification, this peak will become stronger or increase, indicating that carbon–hydrogen bonds increase. The number and intensity of amino absorption bands appearing in the 3000~3500 cm^−1^ region after different polyamine modifications are different. This may be because different polyamines contain different types and quantities of amino groups, such as primary amines, secondary amines, etc. After ethylenediamine modification, the most amino absorption bands appeared in the 3000~3500 cm^−1^ region, indicating that ethylenediamine reacted most fully with PGMA and generated the most amino structures, which can improve the hydrophilicity and chemical stability of adsorbents. Some small peaks appeared in the 1000~1200 cm^−1^ region, which may be caused by the molecular structure of polyamines or reaction by-products. The stretching vibration peak position of carbonyl (C=O) has moved slightly, and new carbonyl peaks have appeared. This may be due to polyamines reacting with carbonyls or forming hydrogen bonds. Two carbonyl peaks appeared after PEI modification due to Fermi resonance. After ethylenediamine modification, a weak absorption peak appeared at 1515 cm^−1^, indicating that fewer carbon–carbon double bonds were consumed after ethylenediamine reacted with PGMA. These carbon–carbon double bonds can maintain the rigidity and porosity of adsorbents and help improve their physical adsorption capacity.

**Figure 3:**
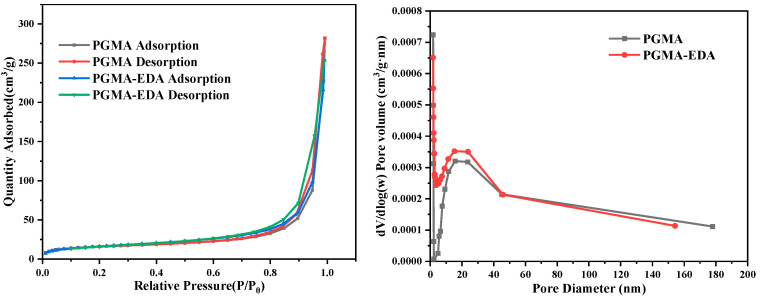
Nitrogen adsorption–desorption curves and pore size distributions of PGMA and PGMA-EDA.

The specific surface area and pore size of PGMA and PGMA-EDA were tested by a surface area analyzer. Their nitrogen adsorption–desorption isotherms are shown in Figure 3. Table 1 shows the specific surface area and average pore size of PGMA and PGMA-EDA adsorbents. It can be seen that the adsorption amount of the material to N_2_ increases with the increase in relative pressure. The physical adsorption isotherm of pure GMA presents a typical IV type and a clear H3 hysteresis loop in the range of 0.6–1.0 P/P0, which has mesoporous structure characteristics. PGMA-EDA is similar to it, indicating that the structure of PGMA is not destroyed after EDA grafting. The specific surface area of pure PGMA is 60.284 m^2^·g^−1^, which is greater than that of PGMA-EDA. This indicates that the specific surface area of the composite material decreases after EDA grafting. In addition, the BJH average pore size of PGMA-EDA is smaller than that of PGMA.

Table 2 shows the element content of PGMA and PGMA-EDA adsorbents. It can be seen that after polyamine grafting, the nitrogen content of PGMA-EDA increased from 0.065% to 4.006%. The change in nitrogen content indicates the successful introduction of polyamine and the successful preparation of PGMA-EDA adsorbent. The presence of amine groups plays a dominant role in the adsorption, and the adsorbent traps Cu(II) through the coordination between amine groups and Cu(II). A higher N content means an increase in the number of amine groups, so complexing polyamines on the substrate can lead to an increase in the adsorption effect. Therefore, PGMA-EDA adsorbent has a greater adsorption capacity for heavy metal ions.

### 2.2. Single-Group Adsorption Behavior

**Figure 4:**
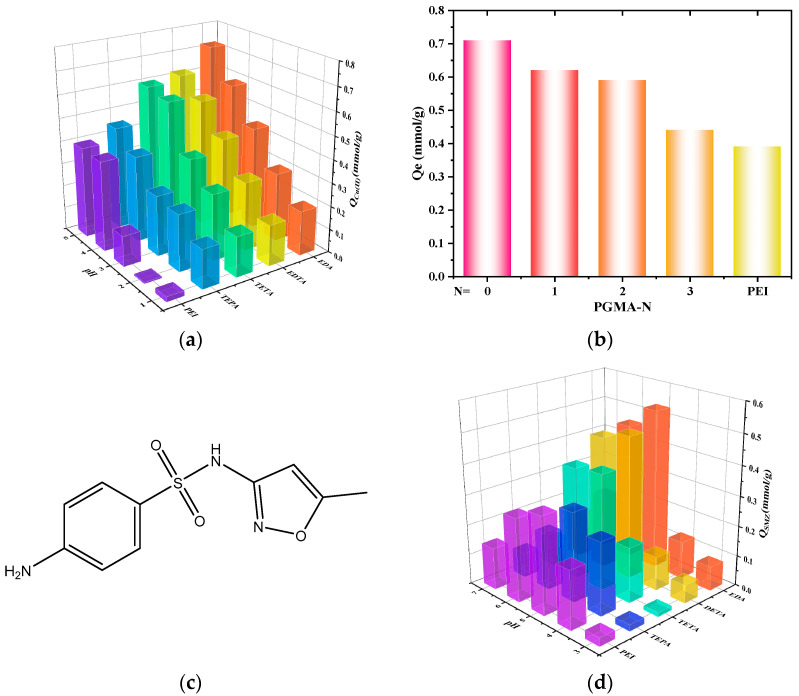
(**a**) The effects of different pH on the effect of adsorption Cu(II) in PGMA-N series absorbers; (**b**) The adsorption effect of PGMA-N during pH = 5; (**c**) The structural format of SMZ; (**d**) The effect of different pH on the effect of adsorption SMZ on the PGMA-N series adsorption agent.

As the pH value changes the degree of ionization of functional groups, the adsorption effect is strongly affected by it. Figure 4a shows the effect of pH value on the adsorption behavior of PGMA-N. Due to the strong bonding tendency between Cu(II) (medium hardness acid) and nitrogen atoms (medium hardness base), PGMA-N adsorbents can have a higher adsorption capacity for Cu(II). Under the condition of pH 5, the adsorption capacity of PGMA-EDA adsorbent for Cu(II) reached 0.72 mmol·g^−1^. As H^+^ concentration increases, adsorption capacity decreases. Moreover, as shown in Figure 4b, it can be observed that the adsorption effect of PGMA-N series adsorbents at pH = 5 increases as the length of the modified polyamine chain becomes shorter. Therefore, in subsequent studies, we mainly focus on PGMA-EDA adsorbent and analyze the adsorption of Cu(II) in weakly acidic and strongly acidic solutions (pH 5 and 1, respectively).

Due to the presence of amino groups in SMZ, as shown in Figure 4c, the pH value of the solution not only affects the charge state of PGMA-N active amino groups but also changes the form of SMZ. Figure 4d shows the adsorption behavior of a single SMZ on PGMA-N. When the pH value increases from 3 to 5, antibiotics gradually change from a positive ion state to an electrically neutral molecular state. When the pH value continues to increase to 7, the electrically neutral molecular state begins to transform into a negative ion state. PGMA-EDA has good adsorption and removal effects on SMZ and is greatly affected by the pH value of the solution in the range of 3~7. The optimal adsorption pH value is 5, and the adsorption amount is 0.51 mmol/g at this time. The interaction between PGMA-N and SMZ mainly occurs through hydrogen bonds. In addition, at pH = 5, part of the amino groups (-NH_2_, -NH-) in PGMA-N are protonated, while a small part of SMZ is in a negative ion state, and part of sulfonamide group (-SO_2_NH-) is deprotonated into -SO_2_N-. PGMA-EDA and SMZ will also produce strong electrostatic forces, making the adsorption amount reach its maximum [38]. When the pH value increases or decreases, both PGMA-N and SMZ will undergo deprotonation or protonation phenomena, and electrostatic forces will be greatly weakened or electrostatic repulsion will occur, resulting in a decrease in adsorption amount. Therefore, we choose pH = 5 as the most suitable for subsequent adsorption experiments.

### 2.3. Co-Adsorption Behavior of Cu(II) and SMZ Composite System

**Figure 5:**
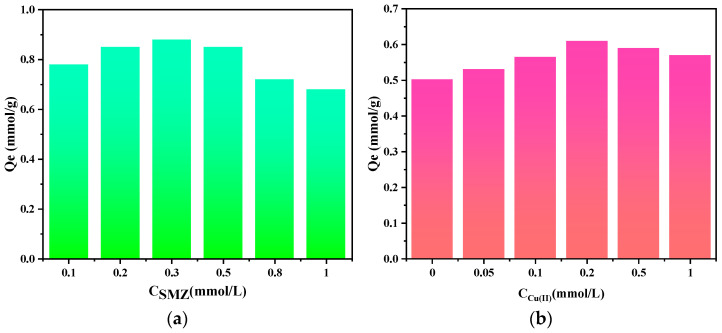
(**a**) The effect of SMZ concentration on the adsorption effect of Cu(II); (**b**) The effect of Cu(II) concentration on the adsorption effect of SMZ.

The influence of SMZ at different concentrations on the removal of Cu(II) and the influence of Cu(II) at different concentrations on the removal of SMZ are shown in Figure 5. As shown in Figure 5a, the adsorption amount of PGMA-EDA on Cu(II) shows a trend of first increasing and then decreasing with the increase in antibiotic concentration, and the change in Cu(II) adsorption amount is most obvious when SMZ coexists. When the concentration of SMZ increases from 0 to 0.3 mmol/L, the adsorption amount of PGMA-EDA on Cu(II) increases from 0.72 mmol/g to 0.88 mmol/g, an increase of 22.2%. Correspondingly, as shown in Figure 5b, when Cu(II) ions are present, the adsorption amount of PGMA-EDA adsorbent on SMZ increases slightly and then decreases after the Cu(II) ion content exceeds 0.2 mmol/L.

The reason for this phenomenon is that the adsorption of antibiotics on the adsorbent is mainly through non-bonded interactions such as hydrophilic and hydrophobic interactions, hydrogen bonds, etc., and the interaction strength is relatively weak. After Cu(II) ions are added, there are two types of competition and co-ligation between Cu(II) ions and antibiotics [39]. When the concentration of Cu(II) is low, Cu(II) in the solid phase may provide new active sites for the adsorption of antibiotics, and bridging dominates, increasing the adsorption amount. After further increasing the concentration, the competitive effect during adsorption gradually increases, and the adsorption amount begins to decrease [40,41]. However, due to the existence of bridging effects, the adsorption amount is always higher than the initial adsorption amount. In addition, at pH = 5, part of SMZ is in a negative ion state and has a salt-promoting effect [42].

### 2.4. Cu(II) Adsorption Isotherms

**Figure 6:**
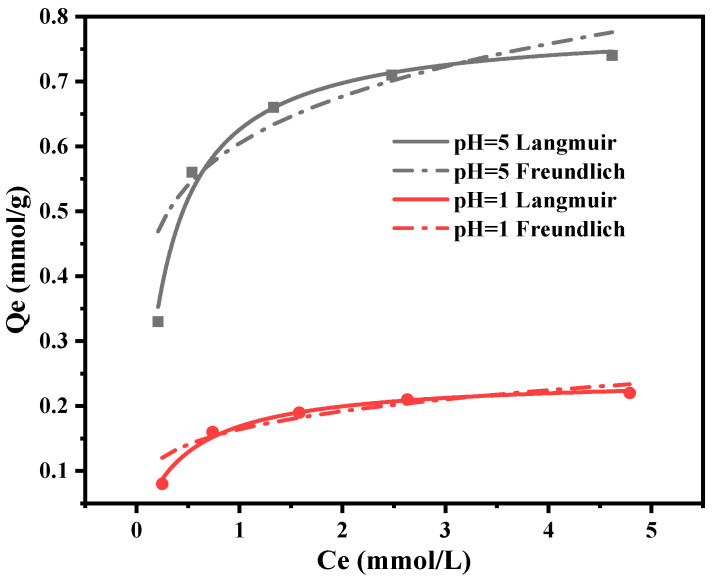
Adsorption isotherms of PGMA-EDA at pH 5 and 1.

The adsorption data of PGMA-EDA on Cu(II) ions at pH 5 and 1 were fitted using the Langmuir and Freundlich models, and the results are shown in Figure 6 and Table 3. The Langmuir model can better fit the data and produce higher correlation coefficients. This indicates that there is a single-molecule layer adsorption process. At pH 5 and 1, the maximum adsorption amounts are 0.794 and 0.244 mmol/g, respectively.

### 2.5. Cu(II) Adsorption Kinetics

**Figure 7:**
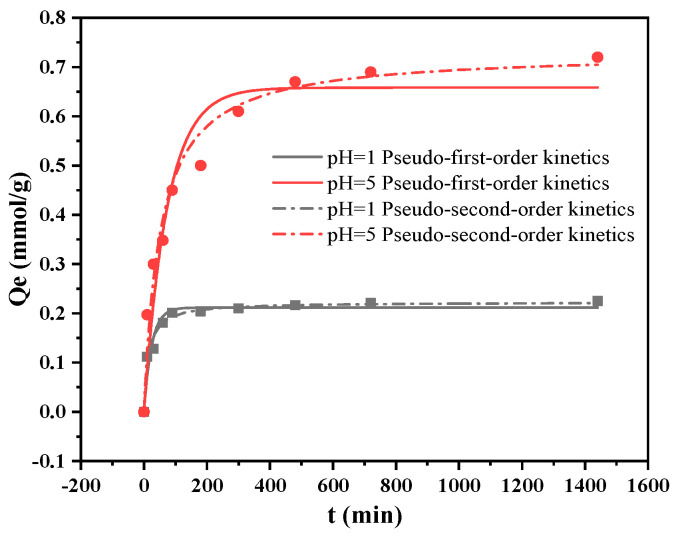
Adsorption kinetics of PGMA-EDA with Cu(II) at pH 5 and 1.

The adsorption kinetics of Cu(II) at pH 5 and 1 are shown in Figure 7 and Table 4. More than 80% of the adsorption occurs within 10 h and reaches equilibrium after 12 h. The kinetic data were simulated by pseudo-first-order and pseudo-second-order models with fitted data, as listed in the table; the pseudo-second-order model can more effectively describe the dynamic adsorption process, indicating that the adsorption process indicated by the adsorbent is dominated by the chemical adsorption, involving ion exchange and functional group interactions.

### 2.6. Adsorption Agent Recycling Performance

**Figure 8:**
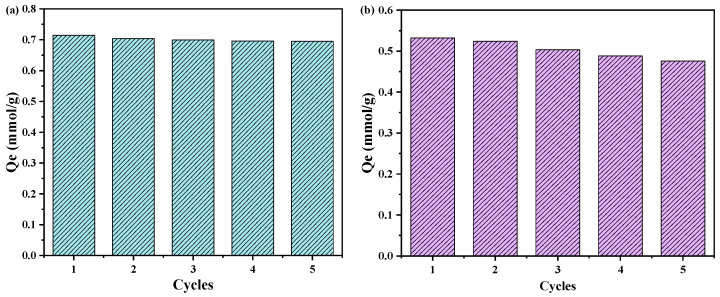
(**a**) 0.3 mol/L HCl’s regeneration and reuse of Cu(II); (**b**) 3 mol/L HCl’s regeneration and reuse of SMZ of PGMA-EDA.

The regeneration and reusability of the adsorbent are crucial in wastewater treatment processes. As is shown in Figure 8a, the exhausted PGMA-EDA containing Cu(II) was regenerated by static desorption with 0.3 mol/L HCl. The results showed that preloaded Cu(II) can be effectively extracted, and the desorption efficiency is higher than 98.5%. Then, the desorbed microspheres were thoroughly rinsed with distilled water until the effluent was neutral. Five consecutive adsorption and regeneration cycles were performed to evaluate reusability, and the adsorption amount of Cu(II) decreased by only 5%, indicating that PGMA-EDA can be effectively reused without any significant capacity loss. As is shown in Figure 8b, during the elution process of antibiotics by 3 mol/L hydrochloric acid, SMZ showed relatively good desorption performance. This is related to the structure of SMZ. Its combination with the adsorbent is mainly through the hydrogen bond between its sulfur and amine groups and the amine groups on the adsorbent. When pickling, these groups will be protonated, and then due to electrostatic repulsion the role of desorption.

### 2.7. Adsorption Mechanism

**Figure 9:**
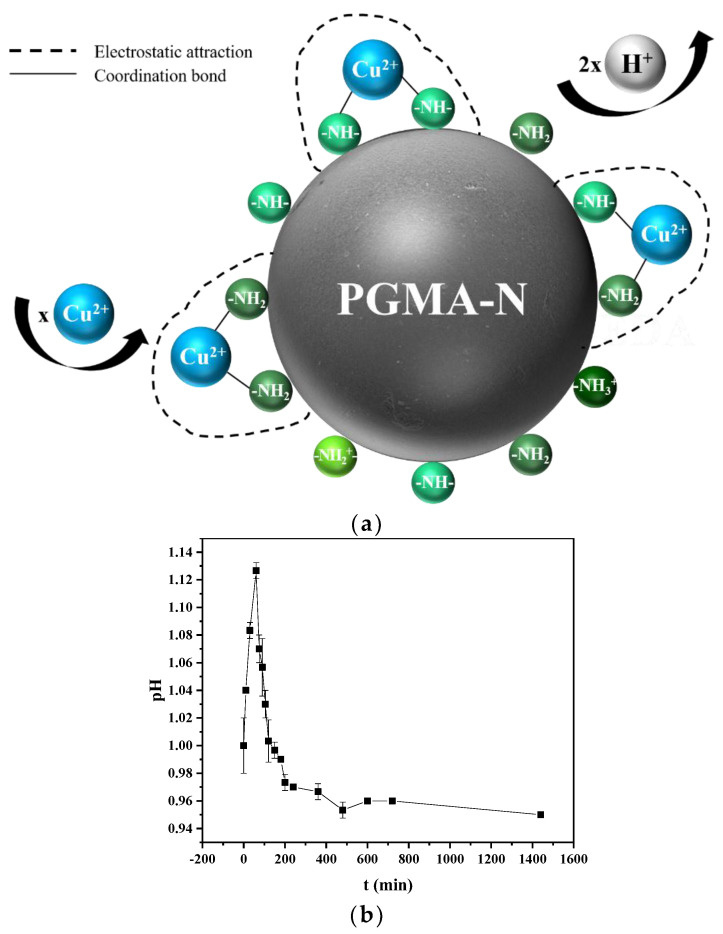
(**a**) Possible mechanism of the adsorption process; (**b**) Time course of pH at the original pH = 1.

As shown in Figure 9b, the adsorption of Cu(II) in a solution with a pH value of 1 shows that H^+^ ions are first rapidly adsorbed by nitrogen atoms, resulting in a slight increase in pH value. Then, the adsorbed H^+^ is displaced by Cu(II) and moved into the bulk solution. Therefore, a decrease in pH value is detected in the latter half. As shown in Figure 9a, the adsorbent PGMA-N can capture Cu(II) through coordination between amino groups and Cu(II). Even at low pH, competitive adsorption between Cu(II) ions and H^+^ can be observed. Therefore, nitrogen atoms of active adsorption sites are partially protonated, resulting in a decrease in adsorption capacity. With the increase in pH value and the decrease in H^+^ concentration, the protonated form of active adsorption sites decreases, thus showing a higher affinity for Cu(II) ions.

There exist more complex forms of interaction between heavy metals and antibiotics, which exhibit a duality of bridging and competitive effects: at low concentrations, both increase adsorption capacity through bridging effects; at high concentrations, competitive adsorption sites lead to a decrease in adsorption capacity, but the total amount of adsorption is still greater than that of single-component adsorption.

## 3. Experimental Section

### 3.1. Materials

The epoxy resin balls were purchased from Xi’an Lanxiao Technology New Materials Co., Ltd., (Xi’an, China), while ethanol, ethylenediamine, diethylenetriamine, triethylenetetramine, tetraethylenepentamine, polyethyleneimine, sodium hydroxide, nitric acid, hydrochloric acid, copper nitrate trihydrate, formic acid, methanol, and sulfamethoxazole were purchased from Shanghai Meryer Chemical Technology Co., Ltd., (Shanghai, China). All chemical reagents are analytical grade and can be used without further purification.

### 3.2. Preparation of PGMA-N Series Adsorbents

**Figure 10:**
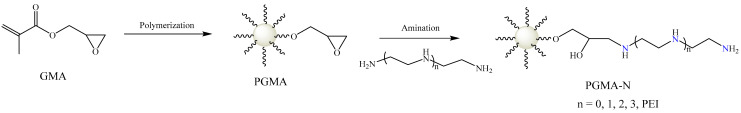
The preparation process of PGMA-N series adsorbents.

Referring to the preparation mechanism of PGMA-DETA by Liu et al. [43], we adopted a simpler method to prepare PGMA-N series adsorbents. In a 500 mL three-necked flask, 20 g of epoxy resin balls were added. At room temperature, 150 mL of polyamine reagents (ethylenediamine, diethylenetriamine, triethylenetetramine, tetraethylenepentamine, polyethyleneimine) were added and allowed to swell for 24 h. The upper clear liquid was then discarded, and 75 mL of corresponding polyamine reagents were added. The mixture was heated to 386 K and stirred at 110 rpm for 24 h. The resulting composite microspheres were extracted with ethanol using a Soxhlet extractor for 4 h to remove impurities in the adsorbent pores. This process produced PGMA-N (N = 0, 1, 2, 3, PEI), which is a methacrylic acid glycol ester chelating adsorbent functionalized with different chain lengths of polyamines. The preparation process is shown in Figure 10.

### 3.3. Characterizations of PGMA-N

The PGMA-N porous adsorbent was characterized using Fourier transform infrared spectroscopy (FT-IR), scanning electron microscopy (SEM), Brunauer–Emmett–Teller (BJH) surface area and pore structure analysis, X-ray photoelectron spectroscopy (XPS), and elemental analysis (EA). The IR spectra were recorded in KBr pellets using a Thermo Scientific Nicolet 6700 Fourier transform infrared spectrometer. Data acquisition and analysis were performed using Omnic software. The morphology of the adsorbent was characterized using a thermal field emission scanning electron microscope (JSM-7100F, JEOL Ltd., Akishima, Japan) at an acceleration voltage of 5.0 kV and a working distance of 6.0 mm. The elemental composition of the adsorbent was analyzed using an elemental analyzer (Vario EL cube, Elementar, Germany). Measurement of specific surface area and mean pore diameter of adsorbents using a Brunauer–Emmett–Teller surface area and pore structure analyzer (ASAP2460, Mack Instruments, Colonial Heights, VA, USA).

### 3.4. Batch Adsorption

Solutions containing antibiotics (sulfamethoxazole) or metal ions (Cu(II)) were prepared separately. All batch experiments were conducted by placing 50 mL of solution in a 100 mL conical flask and then adding 0.025 g of adsorbent into a constant temperature oscillation incubator (Jiangsu Changzhou Nuoji Instrument Co., Ltd., Changzhou, China) for 24 h at 110 rpm and 25 ± 1 °C. The pH value of the solution was adjusted using 0.1 mol L-1 NaOH or HCL solution for metal ions and antibiotics, respectively. After adjustment, the samples were analyzed using an i-Series high-performance liquid chromatography (HPLC) system (Shimadzu Co., Kyoto, Japan) with an Agilent ZORBAX Eclipse Plus-C18 column (150 × 4.6 mm, 5 μm reversed-phase separation column). The liquid phase test conditions were as follows: the mobile phase was a mixed solution of methanol/water (containing 0.1% formic acid) with a volume ratio of 80:20, the detection wavelength was 268 nm, the flow rate was 1 mL/min, and the injection volume was 20 μL. The metal concentration was diluted with 1 mol/L HNO3 and analyzed using an Atomic Absorption Spectrophotometer (AAS) instrument (Shimadzu Co., Kyoto, Japan). All tests were repeated three times, and the amount of adsorbed pollutants was calculated according to the formula:

$$Q_{t}=\frac{\left(C_{0}-C_{t}\right)V}{m}$$

In the formula, Q_t_ (mg g^−1^) represents the amount of adsorbent per gram of adsorbent at time *t* (h). *C*_0_ (mg/L) and *C_t_* (mg/L) represent the initial concentration and residual concentration of adsorbent in the initial solution and filtrate, respectively. *m* (g) and *V* (L) represent the weight of the adsorbent and the volume of the solution.

#### 3.4.1. Influence of Solution pH

This experiment was conducted in batch mode. Hydrochloric acid and sodium hydroxide were used to adjust the initial pH of the model pollutants to different values. The pH values of the Cu(II) solution were 1, 2, 3, 4, and 5, and the pH values of sulfamethoxazole (SMZ) were 3, 4, 5, 6, and 7. Twenty-five milligrams of adsorbent was mixed with 50 mL of Cu(II) (1 mmol/L) or SMZ (0.5 mmol/L) solution adjusted to the desired pH value. The mixture was shaken at a speed of 110 rpm for 24 h at 25 ± 1 °C.

#### 3.4.2. Isothermal Equilibrium Adsorption Behavior

This experiment was conducted in batch mode. Fifty milliliters of adsorbent solution were placed in a 100 mL conical flask, and 25 mg of adsorbent was added. The mixture was shaken for 24 h at 25 ± 1 °C. The initial concentration of Cu(II) was adjusted to 1.0, 2.0, 3.0, 4.0, and 5.0 mmol/L. respectively. The effect of pollutant concentration on adsorption efficiency was studied.

#### 3.4.3. Kinetic Adsorption Behavior

A 250 mg amount of adsorbent was mixed with 500 mL of Cu(II) (1 mmol/L) in a conical flask at pH values of 1.0 and 5.0, respectively; the mixture was shaken at a speed of 110 rpm for 24 h at 25 ± 1 °C. At different time intervals (from ten minutes to twenty-four hours), two milliliters of solution was taken out for analysis.

#### 3.4.4. Influence of Co-Adsorption of Cu(II) and SMZ and Their Mutual Adsorption Effects

A mixed model pollutant of Cu(II) and SMZ was prepared. First, the concentration of Cu(II) (1 mmol/L) was kept constant, and the concentration of SMZ was adjusted to 0, 0.1, 0.2, 0.5, 0.8, and 1 mmol/L, respectively. Then, the concentration of SMZ (0.5 mmol/L) solution was kept constant, and the concentration of Cu(II) was adjusted to 0, 0.05, 0.1, 0.2, 0.5, and 1 mmol/L, respectively. Hydrochloric acid and sodium hydroxide were used to adjust the initial pH of the mixed model pollutant of Cu(II) and SMZ to pH 5.0. Twenty-five milligrams of adsorbent were added to the model pollutant and shaken at a speed of 110 rpm for 24 h at 25 ± 1 °C.

## 4. Conclusions

Herein, PGMA-N series porous adsorbents were prepared by a facile method, which exhibited excellent ability to synergistically remove Cu(II) ions and sulfamethoxazole from aqueous solutions. The amination reaction of the PGMA matrix with polyamines effectively increases the content of amine groups as the main adsorption active sites. During the co-adsorption process, heavy metals and antibiotics showed the duality of bridging and competitive effects. The experimental results showed that the adsorption process of Cu(II) followed the pseudo-second-order kinetic model and Langmuir isotherm. The maximum adsorption capacity of PGMA-EDA for Cu(II) ions was 0.794 mmol/g. In addition, the PGMA-EDA porous adsorbent also demonstrated good stability and reusability after five cycles of regeneration. These results indicate that PGMA-EDA porous adsorbent has great potential for application in treating wastewater coexisting with heavy metals and antibiotics. In water bodies, the coexistence of antibiotics and heavy metals is a serious environmental problem. In this regard, PGMA-EDA can provide an effective and low-cost solution.

## Figures and Tables

**Table 1:** BET surface area, average pore diameter between PGMA and PGMA-EDA.

Adsorption Material Type	BET Surface Area (m^2^/g)	Average Pore Diameter (nm)
PGMA	60.284	34.11
PGMA-EDA	59.4897	24.98

**Table 2:** Elemental compositions of PGMA and PGMA-EDA.

Adsorption Material Type	C (%)	H (%)	N (%)	S (%)
PGMA	60.284	7.226	0.065	0.039
PGMA-EDA	36.175	7.969	4.006	0.052

**Table 3:** Parameters for Langmuir and Freundlich equations of PGMA-EDA with Cu(II) as a function of pH.

Ions	pH	Langmuir Equation			Freundlich Equation		
		*Q_m_/(*mmol·g^−1^*)*	*b*	*R_L_* ^2^	*K_f_*	*n*	*R_F_* ^2^
Cu(II)	5	0.794	0.33	0.988	0.573	4.762	0.859
	1	0.244	1.837	0.990	0.155	3.717	0.876

**Table 4:** Kinetic parameters for the adsorption of PGMA-EDA with Cu(II) at pH 5 and 1.

Ions	pH	Pseudo-Second-Order Kinetics			Pseudo-First-Order Kinetics		
		*k*_2_*/* (g·mmol^−1^·min^−1^)	*Q_e_* (mmol·g^−1^)	*r* ^2^	*k_1_/*min^−1^	*Q_e_/* (mmol·g^−1^)	*r* ^2^
Cu(II)	5	0.0264	0.73	0.971	0.0135	0.658	0.928
	1	0.324	0.223	0.977	0.0411	0.212	0.941

## Data Availability

Not applicable.
